# Supplementary material for: Modeling glioblastoma heterogeneity as a dynamic network of cell states
Source: Mol Syst Biol. 2021 Sep 16;17(9):e10105. doi: 10.15252/msb.202010105 (PMC8444284; doi:10.15252/msb.202010105)
Supplement: Supplementary file 6 — Source Data for Figure 5 [file MSB-17-e10105-s004.zip › Figure5A_sourcedata/GSEA_3017/nosigdb_stateB.GseaPreranked.1622795021725/gsea_report_for_na_pos_1622795021725.html]

Report for na\_pos 1622795021725 [GSEA]

| GS  follow link to MSigDB | GS DETAILS | SIZE | ES | NES | NOM p-val | FDR q-val | FWER p-val | RANK AT MAX | LEADING EDGE || 1 | DOROSZKO\_U3013MG\_CLUSTER\_2 | Details ... | 165 | 0.71 | 4.37 | 0.000 | 0.000 | 0.000 | 194 | tags=73%, list=26%, signal=77% |
| 2 | DALMO\_BARCODENUMBER\_UNPUB | Details ... | 94 | 0.72 | 4.17 | 0.000 | 0.000 | 0.000 | 126 | tags=70%, list=17%, signal=74% |
| 3 | FELDMAN\_2020\_G1 | Details ... | 106 | 0.68 | 3.99 | 0.000 | 0.000 | 0.000 | 155 | tags=69%, list=20%, signal=74% |
| 4 | RICHARDS\_ZHONG\_NATURE\_2018\_NPCS\_UPREG | Details ... | 91 | 0.67 | 3.82 | 0.000 | 0.000 | 0.000 | 186 | tags=75%, list=24%, signal=87% |
| 5 | LARSSON\_STATE\_4 | Details ... | 79 | 0.66 | 3.74 | 0.000 | 0.000 | 0.000 | 171 | tags=72%, list=23%, signal=83% |
| 6 | FELDMAN\_2020\_G2-M | Details ... | 154 | 0.59 | 3.64 | 0.000 | 0.000 | 0.000 | 196 | tags=63%, list=26%, signal=68% |
| 7 | RICHARDS\_NOWAKOWSKI\_SCIENCE\_2017\_MGE\_IPC2\_UPREG | Details ... | 251 | 0.57 | 3.63 | 0.000 | 0.000 | 0.000 | 263 | tags=64%, list=35%, signal=66% |
| 8 | IVY\_CT | Details ... | 78 | 0.65 | 3.60 | 0.000 | 0.000 | 0.000 | 121 | tags=62%, list=16%, signal=66% |
| 9 | FELDMAN\_2020\_S-G2 | Details ... | 172 | 0.56 | 3.54 | 0.000 | 0.000 | 0.000 | 218 | tags=62%, list=29%, signal=67% |
| 10 | GAROFANO\_IAVARONE\_PPR\_SUBTYPE | Details ... | 188 | 0.56 | 3.49 | 0.000 | 0.000 | 0.000 | 267 | tags=69%, list=35%, signal=80% |
| 11 | SEURAT\_CELLCYCLE | Details ... | 72 | 0.62 | 3.48 | 0.000 | 0.000 | 0.000 | 199 | tags=74%, list=26%, signal=90% |
| 12 | RICHARDS\_NOWAKOWSKI\_SCIENCE\_2017\_MGE\_DIV\_UPREG | Details ... | 249 | 0.54 | 3.47 | 0.000 | 0.000 | 0.000 | 284 | tags=66%, list=37%, signal=71% |
| 13 | RICHARDS\_NOWAKOWSKI\_SCIENCE\_2017\_MGE\_IPC1\_UPREG | Details ... | 234 | 0.55 | 3.46 | 0.000 | 0.000 | 0.000 | 270 | tags=65%, list=36%, signal=69% |
| 14 | DOROSZKO\_U3054MG\_CLUSTER\_4 | Details ... | 286 | 0.54 | 3.41 | 0.000 | 0.000 | 0.000 | 277 | tags=62%, list=36%, signal=61% |
| 15 | RICHARDS\_NOWAKOWSKI\_SCIENCE\_2017\_IPC\_DIV1\_UPREG | Details ... | 277 | 0.52 | 3.33 | 0.000 | 0.000 | 0.000 | 196 | tags=49%, list=26%, signal=42% |
| 16 | DOROSZKO\_U3180MG\_CLUSTER\_3 | Details ... | 249 | 0.52 | 3.33 | 0.000 | 0.000 | 0.000 | 284 | tags=64%, list=37%, signal=69% |
| 17 | RICHARDS\_NOWAKOWSKI\_SCIENCE\_2017\_IPC\_DIV2\_UPREG | Details ... | 304 | 0.50 | 3.26 | 0.000 | 0.000 | 0.000 | 268 | tags=58%, list=35%, signal=54% |
| 18 | RICHARDS\_NOWAKOWSKI\_SCIENCE\_2017\_U2\_UPREG | Details ... | 377 | 0.50 | 3.25 | 0.000 | 0.000 | 0.000 | 269 | tags=54%, list=35%, signal=42% |
| 19 | RICHARDS\_NOWAKOWSKI\_SCIENCE\_2017\_RG\_EARLY\_UPREG | Details ... | 128 | 0.52 | 3.12 | 0.000 | 0.000 | 0.000 | 253 | tags=66%, list=33%, signal=82% |
| 20 | NB\_KI\_CLUSTER5 | Details ... | 67 | 0.58 | 3.10 | 0.000 | 0.000 | 0.000 | 243 | tags=76%, list=32%, signal=102% |
| 21 | SUVA\_G2M\_PMID\_31327527 |  | 38 | 0.64 | 3.05 | 0.000 | 0.000 | 0.000 | 156 | tags=74%, list=21%, signal=88% |
| 22 | FELDMAN\_2020\_LATEG1 |  | 48 | 0.59 | 3.01 | 0.000 | 0.000 | 0.000 | 184 | tags=69%, list=24%, signal=85% |
| 23 | RICHARDS\_NEFTEL\_CELL\_2019\_G2\_M |  | 38 | 0.64 | 2.99 | 0.000 | 0.000 | 0.000 | 156 | tags=74%, list=21%, signal=88% |
| 24 | FELDMAN\_2020\_NEURAL G0 |  | 127 | 0.49 | 2.97 | 0.000 | 0.000 | 0.000 | 157 | tags=50%, list=21%, signal=52% |
| 25 | PARADA\_T1\_UP\_26461091 |  | 28 | 0.67 | 2.96 | 0.000 | 0.000 | 0.000 | 116 | tags=68%, list=15%, signal=77% |
| 26 | RICHARDS\_NOWAKOWSKI\_SCIENCE\_2017\_RG\_DIV1\_UPREG |  | 431 | 0.44 | 2.83 | 0.000 | 0.000 | 0.000 | 327 | tags=57%, list=43%, signal=43% |
| 27 | SUVA\_G1S\_PMID\_31327527 |  | 23 | 0.68 | 2.78 | 0.000 | 0.000 | 0.000 | 242 | tags=96%, list=32%, signal=136% |
| 28 | RICHARDS\_NEFTEL\_CELL\_2019\_G1\_S |  | 23 | 0.68 | 2.78 | 0.000 | 0.000 | 0.000 | 242 | tags=96%, list=32%, signal=136% |
| 29 | SINGH\_SOX2\_UPREGULATED\_TRANSCRIPTS\_PMID\_28122245 |  | 67 | 0.49 | 2.70 | 0.000 | 0.000 | 0.000 | 185 | tags=55%, list=24%, signal=67% |
| 30 | PARADA\_T2\_UP\_26461091 |  | 23 | 0.65 | 2.68 | 0.000 | 0.000 | 0.000 | 143 | tags=70%, list=19%, signal=83% |
| 31 | FELDMAN\_2020\_M-EARLYG1 |  | 40 | 0.54 | 2.55 | 0.000 | 0.000 | 0.000 | 157 | tags=58%, list=21%, signal=69% |
| 32 | FELDMAN\_2020\_S |  | 71 | 0.47 | 2.53 | 0.000 | 0.000 | 0.000 | 252 | tags=66%, list=33%, signal=90% |
| 33 | RICHARDS\_CAHOY\_JNEUROSCI\_2016\_CULTURED\_ASTROGLIA |  | 205 | 0.38 | 2.40 | 0.000 | 0.000 | 0.001 | 241 | tags=49%, list=32%, signal=53% |
| 34 | RICHARDS\_CAHOY\_JNEUROSCI\_2010\_ASTRO\_YOUNG |  | 157 | 0.38 | 2.34 | 0.000 | 0.000 | 0.002 | 250 | tags=53%, list=33%, signal=63% |
| 35 | GAROFANO\_IAVARONE\_LIST\_OF\_NEURAL\_PROGENITOR\_MARKER\_GENES |  | 49 | 0.46 | 2.31 | 0.000 | 0.000 | 0.005 | 127 | tags=45%, list=17%, signal=50% |
| 36 | RICHARDS\_NOWAKOWSKI\_SCIENCE\_2017\_MGE\_RG2\_UPREG |  | 118 | 0.38 | 2.25 | 0.000 | 0.000 | 0.008 | 334 | tags=68%, list=44%, signal=102% |
| 37 | DOROSZKO\_U3180MG\_CLUSTER\_4 |  | 50 | 0.42 | 2.13 | 0.000 | 0.000 | 0.028 | 194 | tags=54%, list=26%, signal=68% |
| 38 | RICHARDS\_NOWAKOWSKI\_SCIENCE\_2017\_RG\_DIV2\_UPREG |  | 312 | 0.32 | 2.07 | 0.000 | 0.001 | 0.060 | 326 | tags=55%, list=43%, signal=57% |
| 39 | DOROSZKO\_U3054MG\_CLUSTER\_6 |  | 54 | 0.36 | 1.85 | 0.005 | 0.007 | 0.357 | 194 | tags=46%, list=26%, signal=58% |
| 40 | FELDMAN\_2020\_LOWRNA |  | 144 | 0.30 | 1.81 | 0.002 | 0.009 | 0.455 | 185 | tags=36%, list=24%, signal=39% |
| 41 | ALMSTEDT\_17Q\_MSIGDB |  | 27 | 0.41 | 1.76 | 0.015 | 0.014 | 0.616 | 171 | tags=48%, list=23%, signal=60% |
| 42 | VANGRONINGEN\_ADRN |  | 49 | 0.32 | 1.64 | 0.034 | 0.034 | 0.922 | 325 | tags=65%, list=43%, signal=107% |
| 43 | RICHARDS\_CAHOY\_JNEUROSCI\_2012\_OPC |  | 298 | 0.25 | 1.57 | 0.006 | 0.051 | 0.973 | 300 | tags=47%, list=39%, signal=47% |
| 44 | GANDAL\_AUTISM\_UP\_PMID29439242 |  | 20 | 0.37 | 1.47 | 0.088 | 0.091 | 0.999 | 235 | tags=55%, list=31%, signal=78% |
| 45 | GANDAL\_SCHIZOPHRENIA\_UP\_PMID29439242 |  | 16 | 0.35 | 1.30 | 0.167 | 0.218 | 1.000 | 146 | tags=38%, list=19%, signal=45% |
| 46 | LARSSON\_STATE\_1 |  | 18 | 0.34 | 1.27 | 0.201 | 0.251 | 1.000 | 211 | tags=50%, list=28%, signal=68% |
| 47 | LARSSON\_STATE\_3 |  | 21 | 0.29 | 1.16 | 0.295 | 0.389 | 1.000 | 90 | tags=24%, list=12%, signal=26% |
| 48 | LARSSON\_STATE\_5 |  | 16 | 0.30 | 1.07 | 0.378 | 0.515 | 1.000 | 81 | tags=25%, list=11%, signal=27% |
| 49 | TF\_CENSUS\_TAIPALE |  | 61 | 0.20 | 1.06 | 0.410 | 0.532 | 1.000 | 14 | tags=8%, list=2%, signal=8% |
| 50 | ALMSTEDT\_VGR\_DIFF |  | 97 | 0.18 | 1.05 | 0.423 | 0.531 | 1.000 | 178 | tags=29%, list=23%, signal=33% |
| 51 | OLSEN\_SCPLIKE |  | 16 | 0.27 | 0.97 | 0.511 | 0.658 | 1.000 | 211 | tags=44%, list=28%, signal=59% |
| 52 | KRONA\_ROSEN\_WHITE\_MATTER\_INVASION |  | 24 | 0.22 | 0.94 | 0.530 | 0.682 | 1.000 | 382 | tags=67%, list=50%, signal=130% |
| 53 | KLING\_GBM\_PHENO\_PMID\_27667176 |  | 25 | 0.20 | 0.87 | 0.619 | 0.784 | 1.000 | 191 | tags=32%, list=25%, signal=41% |
| 54 | ALMSTEDT\_11Q\_MSIGDB |  | 33 | 0.18 | 0.80 | 0.695 | 0.868 | 1.000 | 209 | tags=33%, list=28%, signal=44% |
| 55 | RICHARDS\_CAHOY\_JNEUROSCI\_2008\_ASTROCYTE |  | 149 | 0.13 | 0.79 | 0.784 | 0.863 | 1.000 | 226 | tags=31%, list=30%, signal=35% |
| 56 | RICHARDS\_NOWAKOWSKI\_SCIENCE\_2017\_MGE\_IPC3\_UPREG |  | 17 | 0.20 | 0.76 | 0.750 | 0.888 | 1.000 | 280 | tags=47%, list=37%, signal=73% |
| 57 | NB\_KI\_CLUSTER17 |  | 15 | 0.21 | 0.76 | 0.739 | 0.878 | 1.000 | 235 | tags=40%, list=31%, signal=57% |
| 58 | NB\_KI\_CLUSTER16 |  | 16 | 0.19 | 0.68 | 0.846 | 0.943 | 1.000 | 390 | tags=69%, list=51%, signal=138% |
| 59 | OLSEN\_MESENCHYMAL |  | 16 | 0.19 | 0.67 | 0.843 | 0.941 | 1.000 | 389 | tags=63%, list=51%, signal=125% |
| 60 | RICHARDS\_CAHOY\_JNEUROSCI\_2014\_MOG\_POS |  | 37 | 0.14 | 0.66 | 0.863 | 0.936 | 1.000 | 210 | tags=30%, list=28%, signal=39% |
| 61 | GAROFANO\_IAVARONE\_GPM\_SUBTYPE |  | 123 | 0.10 | 0.58 | 0.938 | 0.980 | 1.000 | 642 | tags=91%, list=84%, signal=492% |
| 62 | NB\_KI\_CLUSTER18 |  | 16 | 0.13 | 0.49 | 0.970 | 1.000 | 1.000 | 428 | tags=63%, list=56%, signal=140% |
| 63 | RICHARDS\_NOWAKOWSKI\_SCIENCE\_2017\_CHOROID\_UPREG |  | 64 | 0.08 | 0.41 | 0.996 | 1.000 | 1.000 | 225 | tags=28%, list=30%, signal=37% |
| 64 | RICHARDS\_NOWAKOWSKI\_SCIENCE\_2017\_U3\_UPREG |  | 75 | 0.06 | 0.32 | 1.000 | 1.000 | 1.000 | 91 | tags=11%, list=12%, signal=11% |
Table: Gene sets enriched in phenotype **na **[plain text format]****
